# Correspondence

**Published:** 1864-03

**Authors:** 


### Correspondence.

Messrs. Editors:—Presuming that a report of the Commencement Exercises of the Dental Colleges of this city would interest your readers, I submit the following.

The Annual Commencement of the Pennsylvania College of Dental Surgery, was held at the Musical Fund Hall on the evening of February 26th, 1864, before a large and intelligent audience.

The Graduates number *seventeen*, from a class of *forty-five*, representing ten States, Canada and Cuba, there being four from the latter place.

The Valedictory address was made by Prof. Barker, which was appropriate and well-considered. I append some extracts made in haste and at random, as evidence of its quality.

"The world is filled with the sad evidences of misapplied and misdirected labor. On every hand we witness the heart-broken, despondent and unsuccessful pursuer of some occupation, which, were his talents properly directed would be a shining light in another calling. Our own profession is, unfortunately, not without its representatives of this class, who perhaps struggle along for years, practicing what they do not, and seemingly care not to understand, imperfectly following their calling merely as a means of livelihood, and at last after an aimless life sink into oblivion: no, not oblivion, for they are well remembered, and in sorrow, by unfortunate possessors of ruined dental organisms." * * * *

"In the outset of your career let me entreat you to be stedfast in your honesty of action and of purpose; be not enticed from this by any of those temptations which present so alluring an appearance, and which seem impossible of detection." * * * * *

"Too many, unfortunately, when asked as to the capabilities of such and such a practitioner, can not refrain from some sly inuendo or significant remark which is calculated to convey an unfavorable estimate of his abilities. This is most ungenerous to associates in the same calling, and tends to create in the minds of the public, doubts as to the benefits to be derived from dental surgery and the intelligence of its practitioners." * * * *

"All, however, who claim to be dentists are not thus worthy of your consideration. Thus the unprincipled impostor whose flaming advertisements proclaim how much he has done and can do for suffering humanity, has no such claim; his aim is not how much good he may accomplish, but how much pecuniary benefit he alone may derive. Where such are met with you may caution and advise the unwary, and it is undoubtedly your duty so to do." * * *

"If we look around us, on every side we behold men who without advantages in early life, yet still attain high position. An examination of their career reveals the possession of indomitable perseverance, energy and patience; they are indeed the *irrepressibles*, and they no more succumb to difficulty than the little life-boat which rides so buoyantly the troubled sea, throwing from its tiny sides the swelling wave which at first seems destined to overwhelm, but finally glides away leaving only the dripping spray as an evidence of dangers past."

* * * "In the professional man this season of silent waiting is usually one of considerable trial, but few there are who do not experience it, and the unemployed and leisure hours, if properly devoted to the storing of the mind with the truths of science, may in after years be reverted to as some of the most profitably spent hours of the whole life." * * * * "*Love* for your chosen calling I consider of so much importance, that did you possess all the other qualities and lacked this, I would say, hold! Enter not the sacred portals; you have mistaken your calling, and for your own sake as well as that of others, select some other occupation." * * * *

"There are other qualifications which I have not named, but which are of the first importance, such as agreeable manners and pleasing address. On these qualities will depend in a great degree your future success; possessing these you will be enabled to relieve many of your operations of their painful and disagreeable character: and mildness, patience and judicious sympathy, will not only attract, but retain those who have once been under your treatment." * * * *

"A retrospective glance attests the fact that the age is making constant and increased demands. We see old theories giving way to new, we see enlightened reason, truth and humanity taking their appropriate stations, every thing bears the mark of change—of progress. We can not as a profession expect or wish to be exempt from this progression. * * * Let each one who becomes a dentist, not only constitute himself a *practitioner*, but a *teacher* of the principles of dentistry." * * * * "We therefore say, let every dentist constitute himself a teacher in his own community, let him impart that information to the enquiring which he himself possesses, and which to him has been so freely expounded; let him impress the importance of care, attention, cleanliness, and appropriate treatment when early diseased, of the responsibility resting upon parents to pay proper attention to the temporary teeth of their children." * * * *

"Not all who set out in life with the noblest determinations win this coveted honor. Allurements are on every hand, which tempt successfully the unwary. Thus, many whose early career gave promise of extended usefulness, sink exhausted by the wayside, or toil on dishonoring themselves, and a dishonor to their calling. The professional man, above all others, needs to be ever on his guard. The relation existing between his patients and himself, must necessarily be of an intimate character. He can not hope for, nor expect that relationship, unless his every day life will bear the pure and glorious sunlight. Avoid, therefore, those miscalled pleasures which tend to detract from your good name, and ever remember that you have willingly and cheerfully devoted yourselves to the high and noble duties which you have publicly assumed to-night, and which have for their object the holy mission of alleviating human suffering."

The programmes give the number of patients visiting the clinic of the operative department during the session as twenty-two hundred and two, of whom sixteen hundred and eighty-seven were operated upon, including six hundred and seven gold, and six hundred and ninety tin fillings. Total operations, including extractions, thirty-eight hundred and twenty-eight.

In the mechanical department one hundred and twenty-five patients were supplied with sixteen hundred and forty-seven artificial teeth, comprising twenty-one full sets, forty-two full upper sets, and three full lower, including blocks, continuous gum, metal and hard rubber plates.

The First Annual Commencement of the Philadelphia Dental College, was held at Concert Hall, on the evening of February 29th, 1864, and which attracted a large and enthusiastic audience.

The graduates were *six* in number, from a class of *fifteen.* The charge to the graduating class was delivered by Prof. Kingsbury, which address, although very entertaining and peculiarly appropriate to a mixed or non-professional audience, was a little too general in its scope, and a good deal too long in its delivery for the time and the occasion. There were many good points in it that I would much like to give had I access to the address.

The programme states that the operations in the operative department number ten hundred and sixty-six. In the mechanical department the whole number of teeth mounted were eight hundred and ninety-eight.

Trusting you have had a favorable season in your College, in Cincinnati, I am Yours,

PHILAD., March 12, 1864. O. U. C.

